# Chronic In Vivo CRISPR-Cas Genome Editing: Challenges, Long-Term Safety, and Outlook

**DOI:** 10.3390/cells15020156

**Published:** 2026-01-15

**Authors:** Caroline Bao, Catherine I. Channell, Yi Hsuan Tseng, Johnathan Bailey, Naeem Sbaiti, Aykut Demirkol, Stephen H. Tsang

**Affiliations:** 1 Department of Ophthalmology, Columbia University Irving Medical Center, New York, NY 10032, USA; cic2124@cumc.columbia.edu (C.I.C.); jab2477@cumc.columbia.edu (J.B.); ns3918@cumc.columbia.edu (N.S.); ademirkol79@hotmail.com (A.D.); sht2@cumc.columbia.edu (S.H.T.); 2 Department of Medicine, Mackay Medical University, New Taipei City 252, Taiwan; 110910028@live.mmu.edu.tw; 3 Jonas Children’s Vision Care, and Bernard & Shirlee Brown Glaucoma Laboratory, Department of Ophthalmology, Columbia University, New York, NY 10032, USA

**Keywords:** CRISPR/Cas, gene therapy, genome editing, therapeutics, clinical trials, off-target effects

## Abstract

CRISPR/Cas systems have transformed molecular medicine, yet the field still lacks principled guidance on when transient editing suffices versus when sustained exposure through in vivo viral delivery is necessary and how to keep prolonged exposure safe. Notably, EDIT-101 was designed for a permanent edit in post-mitotic photoreceptors with lifelong Cas9 persistence. This review addresses this gap by defining the biological and therapeutic conditions that drive benefit from extended Cas activity while minimizing risk. We will (i) examine relationships between expression window and efficacy across Cas9/Cas12/Cas13 modalities, (ii) identify genome-wide off-target liabilities alongside orthogonal assays, and (iii) discuss controllable, self-limiting, and recallable editor platforms. By separating durable edits from persistent nuclease exposure, and by providing validated control levers, this work establishes a generalizable framework for safe, higher-efficacy CRISPR medicines. Furthermore, we highlight key studies in cell lines, murine models, non-human primates, and humans that examine the long-term effects of sustained expression of CRISPR/Cas systems and discuss the safety and efficacy of such approaches. Current evidence demonstrates promising therapeutic outcomes with manageable safety profiles, although there is a need for continued monitoring as CRISPR/Cas therapies are increasingly applied in clinical contexts and therapies are developed for broader clinical applications.

## 1. Introduction

The CRISPR/Cas system, originally discovered as a microbial adaptive immune mechanism, has revolutionized modern molecular biology through its repurposing as a tool for precise gene editing. Clustered regularly interspaced short palindromic repeats (CRISPR)-associated (Cas) proteins, particularly Cas9, Cas12, and Cas13, have emerged as central effectors in diverse applications ranging from functional genomics to therapeutic gene correction. While short-term gene editing outcomes have been extensively studied, increasing attention is being directed toward the long-term safety, efficacy, and cellular consequences of chronic Cas protein expression, especially in the context of in vivo therapeutic delivery.

This review focuses on the evolving understanding of Cas protein biology, therapeutic strategies leveraging their unique enzymatic properties, and the systemic implications of their prolonged activity. We explore the regulatory mechanisms controlling *cas* gene expression in prokaryotes, distinctions among various Cas types and their delivery methods, and the challenges associated with persistent endonuclease activity, including double-stranded break repair, off-target effects, and immunogenicity. By synthesizing findings from cell lines, animal models, and human clinical trials, we highlight critical insights into the long-term safety of CRISPR-based interventions and emerging modifications designed to enhance safety and control in therapeutic contexts.

## 2. Cas Proteins

Cas proteins play a central role in the adaptive immune system found in many bacteria and archaea. This structure was first reported by Ishino in 1987 [1] and Jansen later proposed the acronym CRISPR in 2002 [2]. After hyper-variable spacers with sequences similar to foreign plasmids and viruses were discovered in 2005, Mojica et al. proposed the structure’s role in protecting against transmissible genetic elements [3]. Since then, additional research, discoveries, and reviews have expanded on the known role of Cas proteins. 

### 2.1. Intrinsic Role of Cas Proteins in Prokaryotes

It is now understood that the CRISPR/Cas system has evolved in prokaryotes as part of a defense mechanism against invading viruses or foreign genetic materials. When a bacterium survives a viral infection, it integrates small parts of viral DNA called protospacers into its genome within CRISPR loci [4]. The new protospacer-CRISPR sequence is then transcribed into small CRISPR RNAs (crRNA) [5]. When the same virus tries to infect again, the crRNA guides the Cas protein to the matching viral DNA, which is then cleaved and neutralized [5,6,7]. In this way, the CRISPR arrays become a record of previous infections, and the CRISPR/Cas system is able to protect against foreign genetic material. 

### 2.2. Expression of Cas Proteins in Prokaryotes

As important but metabolically expensive elements in the adaptive immune system of prokaryotes, Cas proteins are only activated under specific conditions, and natural regulation largely falls into several general patterns [8]. These internal and external cues allow for activation or repression of *cas* genes. 

One such pattern is cell density, where CRISPR/Cas elements of bacteria without biofilm become upregulated in response to the higher density and their subsequent increased susceptibility to viral infections and horizontal gene transfer [8,9]. *Cas* gene expression can also vary in response to different levels of metabolic activity. Depending on the species of bacteria and the subsequent location of CAP binding sites, the cAMP-CAP complex can either activate, such as in *P. atrosepticum*, or repress *cas* gene expression, like in *E. coli*, in direct response to glucose deficiency [10,11]. CRISPR/Cas is also regulated by NAPs or nucleoid-associated proteins such as H-NS, StpA, and LRP, which downregulate activity by inhibiting gene transcription via strong condensation of the DNA [8].

### 2.3. Use of Cas Proteins Therapeutically

As the molecular understanding of Cas protein regulation in prokaryotes deepens, researchers have begun to adapt this knowledge to harnessing CRISPR/Cas systems for therapeutic purposes. The precision and programmability of Cas proteins have made them useful candidates for correcting genetic mutations that cause a range of inherited and acquired diseases. By mimicking the natural targeting mechanism of crRNA-guided cleavage, scientists can direct Cas proteins to specific genomic loci in human cells, enabling therapeutic interventions at the DNA or RNA level. Therapeutic applications typically involve guiding Cas proteins to these targets using engineered RNA molecules, allowing for the disruption, correction, or modulation of disease-related genes. 

The successful translation of CRISPR/Cas systems into therapeutic uses depends not only on the editing mechanism itself but also on the choice of Cas protein and its delivery method. Different Cas proteins exhibit unique targeting capabilities and biochemical properties that influence the therapeutic potential and safety profile of the intervention. As the next section details, understanding the distinctions between different CRISPR/Cas systems is critical for designing strategies that maximize efficacy while minimizing risks such as off-target effects and immunogenicity. 

Additionally, long-term durability remains a central challenge for in vivo genetic therapies. For example, Luxturna (voretigene neparvovec), an AAV-mediated gene therapy that restores gene function through episomal transgene expression, has demonstrated meaningful visual benefit; however, long-term follow-up and post-marketing observations indicate that retinal degeneration may continue in some patients years after treatment [12]. These findings suggest that therapeutic efficacy may be limited by the inability of gene addition alone to permanently modify disease-driving genetic defects or by declining functional transgene expression over time. In this context, in vivo CRISPR/Cas-based approaches that enable sustained or repeated genomic correction through longer term Cas expression may offer a potential advantage for achieving more durable disease modification, while acknowledging that such strategies introduce distinct safety and regulatory considerations that necessitate longer term follow-up.

## 3. Types of Cas Proteins, Engineering Modifications and Their Delivery

CRISPR/Cas systems are categorized into two classes (Class 1 and Class 2), based on the structure of their effector complexes. Class 1 systems utilize a multi-Cas protein complex and include type I, III, and IV, employing representative endonucleases of Cas3, Cas10, and DinG, respectively. Class 2 systems use single proteins that encompass type II, V, and VI, cleaving the genetic code with Cas9, Cas12–Cas14, and Cas13, respectively. Among these, type I, II, and V target DNA, type III targets both DNA and RNA, and type VI exclusively targets RNA [13,14,15]. Recent advances have centered around Class 2 effectors, like Cas9, Cas12, and Cas13, which have been extensively researched as molecular diagnostic and therapeutic tools.

### 3.1. Cas9

Cas9, a type II Class 2 nuclease initially derived from Streptococcus pyogenes, is the first and most in-depth researched nuclease and has been widely used to edit DNA. Cas9 recognizes a protospacer adjacent motif (PAM), typically 5’-NGG [16] and complexes with both natural guide RNA (gRNA) and modified single-guide RNA (sgRNA) [17]. First used by Gasiunas et al. [18], gRNA was refined by Jinek et al. by fusing crRNA and trans-activating CRISPR RNA (tracrRNA) into a sgRNA. This sgRNA plays a crucial role in providing target guidance for the Cas9 system cleavage. 

Substantial efforts have been devoted to engineering Cas9 proteins and guide RNAs to address core limitations in genome editing, such as off-target effects, genotoxicity associated with double-strand breaks (DSBs), and constraints on the targeting scope [14,19].

To mitigate off-target effects caused by non-specific DNA binding, high-fidelity variants have been developed. These include enhanced specific SpCas9 (eSpCas9) and SpCas9-High-Fidelity variant #1, retaining high on-target activity, while simultaneously weakening non-specific interactions [20,21]. Moreover, to temporarily limit Cas9 activity and thereby reduce the off-target risk associated with chronic expression, the anti-CRISPR protein, such as AcrIIA4, has been utilized to suppress Cas9 function after delivery [22]. Similarly, sgRNA engineering, specifically refining the scaffold structure, has been shown to improve target binding fidelity and reduce off-target activity [23].

Concerns regarding genotoxicity from DSBs were also addressed. Adapting Cas9 into base editors and prime editors enables precise nucleotide conversions or small insertions/deletions without inducing DSBs [24]. The catalytically inactive variant, dead Cas9 (dCas9), lacks DNA cleavage activity, while its DNA binding activity remains unaffected. It is further fused with CRISPR activation (CRISPRa) or interference (CRISPRi), which enables activation or inhibition of the target gene, respectively [14,25], thereby completely avoiding the creation of DSBs. Editing technology has also been developed for more durable epigenetic editing. The CRISPRon and CRISPRoff system rely on a dCas9 enzyme fused to a ten-eleven translocation (TET) DNA demethylase catalytic domain or DNA methyltransferase, respectively, to establish heritable epigenetic changes [26,27]. Such epigenetic modifications avoid some of the mutational risk of introducing an active Cas enzyme but are more limited in the range of effects.

Finally, the inherent restriction imposed by the PAM sequence requirements has been overcome by developing engineered variants, such as xCas9 and SpRY, which exhibit relaxed PAM recognition capabilities, consequently broadening the accessible genome-targeting range [23]. In parallel, sgRNA engineering has also advanced to improve specificity and efficiency. Refining the sgRNA structure has been shown to boost editing efficiency in systems with limited vector capacity [28]. Synthetic synthesis of sgRNAs show lowering the host immune response compared to in vitro transcribed sgRNAs [29]. Together, these Cas9 and sgRNA innovations enhance the safety, precision, and applicability of CRISPR-based genome engineering.

### 3.2. Cas12

Cas12 (type V), like Cas9, is an RNA-guided DNA endonuclease. Cas12 utilizes a single crRNA and recognizes a T-rich PAM [30]. However, there are distinct differences between them in RNA processing, DNA targeting binding and catalytic activity. The staggered end created by Cas12 cleavage can facilitate more precise integration of foreign DNA into the genome [31].

Despite these distinct advantages, the therapeutic application of Cas12 is often constrained by its strict PAM sequence preference (typically 5’-TTTV), variable activity across cell types, and relatively lower editing efficiency in mammalian systems. To overcome these limitations, a range of Cas12 engineering efforts have been pursued.

A primary area of modification has been expanding the targeting scope and improving specificity. Engineered variants such as enAsCas12a and enLbCas12a (enhanced Acidaminococcus and Lachnospiraceae Cas12a, respectively) have been developed to exhibit relaxed PAM recognition, enabling the targeting of non-canonical sites while retaining strong on-target activity [32]. Furthermore, modifications to the crRNA itself have increased binding affinity and stability, such as in zCRISPR-Cas12a, incorporating 2-aminoadenine (base Z) into the crRNA, but still preserves the low off-target profile [33]. Research demonstrates that Cas12a effectors possess a high degree of conformational adaptability, allowing them to recruit crRNAs with noncanonical features such as loop extensions and split scaffolds. ErCas12a (also known as MAD7) has emerged as a particularly versatile effector [34].

In addition to guide RNA refinements, new Cas12 variants address delivery and specificity challenges. The development of hypercompact variants like Cas12f (also known as Cas14) offers a significant advantage due to its small size, which is less than half that of Cas12a variants. This facilitates efficient packaging into non-viral delivery systems. Cas12f is also noted for its high specificity and a reduced propensity for chromosomal translocations or large deletions [35]. To broaden the application scope, variants originally restricted by temperature limitations, such as wild-type Cas12b, have been optimized into engineered forms like BhCas12b v4, achieving robust function at physiological temperatures (37 °C) for mammalian genome editing [36]. 

### 3.3. Cas13

Cas13 (type VI) functions as an RNA-guided endonuclease targeting single-stranded RNA (ssRNA). Crucially, by modifying RNA rather than DNA, Cas13 avoids permanent genomic changes [37]. Cas13 has a relaxed or no requirement for a protospacer-flanking site, thereby expanding its targeting flexibility compared to Cas9 and Cas12 [38]. 

The Cas13 protein is a single-subunit RNA-guided enzyme, consisting of recognition (REC) and nuclease (NUC) lobes. The NUC lobe contains two Higher Eukaryotes and Prokaryotes Nucleotide-binding (HEPN) domains, contributing to target RNA accommodation and subsequent catalytic cleavage. Cas13 possesses inherent endoribonuclease activity, enabling the maturation of its own precursor crRNA (pre-crRNA) into the functional guide crRNA [38]. Upon binding of the Cas13-crRNA complex to a complementary target ssRNA, significant conformational changes are induced, leading to the activation of the HEPN catalytic pocket. This activation results in two linked activities: sequence-specific cleavage of the target RNA (cis-RNase activity) and indiscriminate collateral degradation of nearby nonspecific ssRNA molecules (trans-RNase activity) [39]. This collateral effect is exploited in highly sensitive nucleic acid detection platforms [39]. Cas13 holds potential for transient gene regulation, RNA knockdown, and viral RNA targeting.

Engineering efforts have addressed limitations in Cas13’s efficiency, specificity, and collateral cleavage activity across its numerous subtypes (Cas13a, 13b, 13c, 13d, 13j, and 13X/Y) [40,41]. To minimize bystander RNA degradation, high-fidelity nucleases have been developed through the mutagenesis of variants such as Cas13d and Cas13X, which are optimized for strong on-target activity with reduced collateral cleavage [42]. Catalytically inactive Cas13 (dCas13), a non-degrading application has been developed. Fusion of dCas13 with deaminases allow for single-nucleotide RNA base editing, with continuous optimization reducing off-target effects [43]. Furthermore, dCas13 has been functionally adapted for applications including splicing modulation, programmable N6-methyladenosine (m6A) editing [44], and translational repression by blocking ribosome binding [45,46]. Both active Cas13 and dCas13 enable sensitive detection of nucleic acids, particularly RNA, through fluorescence, lateral flow, and electrochemical signals, used widely in the detection of viruses [47]. A recent advance is the photoactivatable RNA base editor, combining a compact dCas13X scaffold with a split ADAR2 deaminase domain. Its small size allows AAV delivery, and its light-inducible activation provides precise spatiotemporal control, thereby reducing overall off-target activity. This inducible system also offers opportunities for multiplexed and reversible regulation, such as enhancing immune cell persistence and function in CAR-T therapies [48].

## 4. Therapeutic Delivery Systems

Efficient and safe delivery of CRISPR/Cas components is a central challenge for successful genome editing, as delivery modality directly influences editing efficiency, persistence, tissue specificity, and safety outcomes. Delivery methods are broadly categorized into physical methods, viral vectors, and non-viral vectors [14]. Physical methods, including electroporation, microinjection and mechanical cell deformation, rely on direct force to facilitate intracellular delivery and are primarily used in in vitro experiments [14].

Viral vectors remain the most widely used delivery method due to their high transduction efficiency and ability to sustain expression. Among them, adeno-associated viruses (AAVs) are the most common vectors, offering long-term gene expression and broad tissue tropism. However, AAVs are limited by their small cargo capacity (4.7kb) and potential to induce immune responses [49,50]. Lentiviral vectors, another common choice, have a larger cargo capacity but integrate into the host genome and may increase the risk of insertional mutagenesis and off-target effects [51]. Adenoviral vectors (AdVs) are also utilized for CRISPR component delivery, particularly in ex vivo settings using patient primary cells or established cell lines. It has a larger package capacity and transient pattern for its non-integrating function [52]. However, it also possesses the immunogenicity problems, that around 90% of the human population has pre-existing antibodies against AdV [31]. Despite these challenges, viral vectors remain central to clinical translation, exemplified by AAV-based EDIT-101 for LCA.

Non-viral vectors, including lipid nanoparticles (LNPs), polymers, inorganic nanoparticles, and ribonucleoprotein (RNP) complexes, are delivery systems for gene and nucleic acid therapeutics with short-lived character. Compared to viral vectors, non-viral vectors generally have lower delivery efficiency and less robust tissue tropism, but offer superior safety profiles: reduced immunogenicity, no risk of insertional mutagenesis, and easier scalability [53]. LNPs are the most clinically advanced non-viral vectors and demonstrated efficient encapsulation of Cas mRNA and guide RNAs, enabling transient expression with reduced integration risk [54]. NTLA-2001, which uses an LNP to deliver mRNA encoding Cas9 and a guide RNA targeting the TTR gene for in vivo CRISPR/Cas9 gene editing in patients with hereditary transthyretin amyloidosis, showed long-lasting TTR reduction in a phase 1 clinical trial [55]. RNP complexes, directly delivering pre-assembled Cas nuclease protein with guide RNA, provide transient genome editing with minimal off-target effects and reduced immunogenicity [53]. N-acetylgalactosamine (GalNAc) is the most used small nucleic acid drug delivery system. Several drugs using GalNAc as a delivery system have been approved as therapy for diseases, such as acute hepatic porphyria, primary hyperoxaluria type 1, and polyneuropathy in adults with hATTR amyloidosis [56].

The differences between transient and sustained delivered systems are summarized in Table 1.

The persistence of different delivery systems for CRISPR/Cas genome editing in humans varies significantly by modality. Viral vectors persist for years, synthetic non-viral systems persist for days, plasmid DNA can persist for weeks to months in human cells, and LNPs and RNP complexes are degraded within hours to days [57]. Long-term expression from viral vectors can benefit durable gene therapies but increases risks of genotoxicity and immune reactions. In contrast, transient systems such as LNPs and RNPs favor short-lived yet precise interventions [56]. Future research should focus on optimizing vector design to improve their transfection efficiency and targeting capabilities.

## 5. Long-Term Complications of CRISPR Expression

Therapies incorporating Cas9 expression have emerged as powerful tools for targeted genome editing, holding significant promise for treating genetic disorders. Despite their potential, safety concerns may complicate therapeutic applications. Adverse effects of CRISPR/Cas expression are summarized in Table 2. AAV vectors, the most common delivery method in current clinical trials, can maintain transgene expression for more than a decade in human tissues, causing concern that Cas endonucleases introduced could remain active for a similar period of time [58]. Such length of expression makes it essential to understand the full spectrum of CRISPR/Cas activity that could occur during chronic exposure to endonuclease activity.

### 5.1. Double Stranded Breaks

Endonuclease activity of CRISPR/Cas9 and Cas12 systems forms DSBs, which are repaired either through HDR or one of several end joining pathways [59]. Cas9 and Cas12 exhibit key mechanistic differences in how they generate DSBs and process RNA. 

Cas9 uses two distinct domains to cleave both DNA strands. HNH cleaves the DNA strand complementary to the guide RNA, and RuvC cleaves the non-target DNA strand [60]. This dual-domain action leads to a blunt end DSB, and is preferentially handled by the Non-Homologous End Joining (NHEJ) pathway. Cas12 contains a single RuvC domain, which cleaves both single-stranded or double stranded DNA. This action produces a staggered-end DSB promoting the use of the Homology-Directed Repair (HDR) pathway [60].

HDR is a method of DNA repair that involves resection at the DSB followed by DNA synthesis on a donor template, either an exogenous DNA strand introduced to the cell or the sister chromatid, resulting in accurate repair [61]. 

In contrast, NHEJ is the most common repair pathway in many cells, occurring most commonly during the S and G2 phases of the cell cycle and acts rapidly to suppress chromosomal translocations [62,63]. Following the formation of a DSB by a Cas endonuclease, the Ku70-Ku80 (Ku) heterodimer first recognizes the presence of DSBs, binding to the synapse to recruit DNA-dependent protein kinase catalytic subunits (DNA-PKcs) and form the DNA-PKcs complex. In Cas9 editing, which results in blunt end cuts, repair of DSBs through NHEJ occurs immediately after formation of the DNA-PKcs complex by the Ligase IV/XRCC4 complex [64]. Cas12 endonuclease activity creates a 5’ overhang, recruiting the endonuclease Artemis to complex with DNA-PKcs and cleave the single stranded region of DNA to form a blunt ended DSB, which may result in small deletions [65]. 

Microhomology mediated end joining (MMEJ) and single stranded annealing (SSA) are two other repair pathways that involve annealing of short, complementary homologies followed by DNA synthesis. These mechanisms are intrinsically mutagenic and frequently result in insertions and deletions (indels) [66,67]. 

Chromosomal translocations have been attributed to both NHEJ and MMEJ, suggesting that, beyond indels, end joining repair pathways are prone to introducing larger genomic rearrangements [68]. While such rearrangements theoretically have a risk of being oncogenic, follow-up across CRISPR clinical trials and observational cohorts has not yet revealed tumor formation attributable to therapeutic CRISPR editing, though continued surveillance is warranted [69,70].

### 5.2. Off-Target Genomic Editing

Beyond the intended loci, CRISPR/Cas systems can bind and cleave at sites containing several mismatches to the guide RNA. While correct base pairing at the seed sequence in the sgRNA, which includes the first few base pairs next to the PAM site, is required for binding and subsequent enzymatic activity, mismatches are tolerated more distally to the PAM site [71,72,73]. Biochemical profiling shows that sequences carrying up to seven mismatches outside of the seed region can be cut in vitro with similar efficiency to the target site [17]. In vivo, chromatin accessibility restricts off-target nuclease activity; heterochromatin or tightly packed regions diminish off-target cleavage [74,75,76].

The duration of Cas protein expression may also affect off-target editing. In a doxycycline-inducible system, on-target editing reached a plateau within days, whereas off-target mutations continued to accumulate throughout a two-week experiment, demonstrating that prolonged nuclease expression disproportionately magnifies off-target burden [77]. Multiple approaches, including redesign of sgRNA structure, use of off-target prediction in sgRNA design, and engineering of more high fidelity Cas proteins, have been taken to decrease the likelihood of off-target editing and will be discussed in subsequent sections. 

### 5.3. Off-Target Transcriptomic Effects

Non-specific off-target gRNA binding may also result in unintended edits to the transcriptome. Certain CRISPR-based platforms have been shown to induce widespread RNA alterations in addition to DNA editing. One 2019 study found that a class of cytosine-targeting base editors caused tens of thousands of cytosine-to-uracil changes across the transcriptome [78]. However, improved design, including increased nuclease specificity and more specific gRNA design, greatly reduced the number of RNA edits, demonstrating that it is possible to reduce the transcriptome-wide effect of such editors [78].

Beyond direct edits to the transcriptome, other effects of off-target DNA targeting may also cause more global transcriptomic changes. For one, transcriptional modulation techniques such as CRISPRi may result in changes to the transcriptome when gRNAs bind to sequences nonspecific to the target sequence [79]. Furthermore, off-target CRISPR/Cas9 activity at transcriptional features may also result in unintended alterations to gene expression [80]. 

### 5.4. Genomic Vector Integration

When delivered as part of an AAV vector, Cas9-induced DSBs can promote integration of the AAV genome into the host DNA. While AAV vectors generally remain as episomes within the cell, integration has been observed into chromosomal DNA in the process of DSB repair [81,82,83,84]. More recent research has found that concatemeric insertions can occur with use of AAV6 vectors, which may not be readily detected via PCR, result in inconsistent levels of target gene transcription, and affect transcriptional regulation of other genes [85]. 

Although these integration rates raise concerns about insertional mutagenesis, preclinical and clinical data suggest that the associated risk may be limited. To date, no genotoxic effects have been reported across multiple clinical trials involving AAV-based gene therapies [84]. Targeted genomic analyses further support this, showing minimal integration and no enrichment at oncogenic loci. In one preclinical study, AAV1 vectors encoding *Streptococcus pyogenes* Cas9 (SpCas9) and guide RNAs targeting three therapeutic genes were delivered via intrahippocampal injection to cynomolgus monkeys and mice and monitored for 6 and 8 weeks, respectively. Although some preferential integration occurred within transcriptional units, no enrichment was detected near cancer-associated genes [81]. Another study examining liver biopsies from both patients and nonhuman primates enrolled in a gene therapy trial for acute intermittent porphyria found 7.44  ×  10^−5^ and 1.00  ×  10^−4^ integration sites per cell in primates and 2.00  ×  10^−4^ and 1.17  ×  10^−3^ integration sites per cell in humans. Importantly, none of these integrations occurred at sites linked to hepatocellular carcinoma, indicating a lack of genotoxicity in both preclinical models and human subjects [84]. Thus, while AAV integration at DSBs is a safety consideration, current evidence suggests the oncogenic risk is low. 

### 5.5. Immunogenicity

CRISPR/Cas therapeutics can elicit immune responses due to the introduction of foreign proteins, the presence of sgRNAs, or the vectors used for delivery. Managing these immune responses is essential to minimize adverse effects and ensure therapeutic efficacy. 

Immune responses have been observed to occur against both the Cas proteins and sgRNAs. Cas proteins, such as SpCas9 and *Staphylococcus aureus* Cas9 (SaCas9), are derived from organisms that commonly colonize or infect humans, making them potential targets of pre-existing immunity [86]. Adaptive immune responses to Cas9 and Cas13 orthologs have been detected in healthy individuals, as demonstrated by the presence of specific antibodies and reactive T cells in in vitro assays [87,88,89]. In addition to protein components, sgRNAs produced by in vitro transcription can activate innate immune sensors, particularly those recognizing PAMPs [90]. Furthermore, methods of CRISPR/Cas delivery, which include both viral vectors and non-viral delivery methods, can activate different immune sensors. AAVs, which are the most common method of delivery for CRISPR/Cas therapeutics, stimulate both the adaptive and innate immune system, albeit to a lesser degree than most other viruses [91]. Immune responses to low to moderate doses of AAVs are generally mild, commonly failing to stimulate detectable T cell responses [92]. At higher doses, however, adverse events such as death resulting from hyperactivation of the innate immune system, thrombotic microangiopathies, and hemolytic uremic syndrome have been reported [93,94,95]. LNPs were found to have lower immunogenicity based on mouse trials and allow for repeated injection, which is not possible with AAVs due to the development of neutralizing antibodies [96]. LNP application, however, has been more limited thus far than AAVs in human trials.

**Figure 1:**
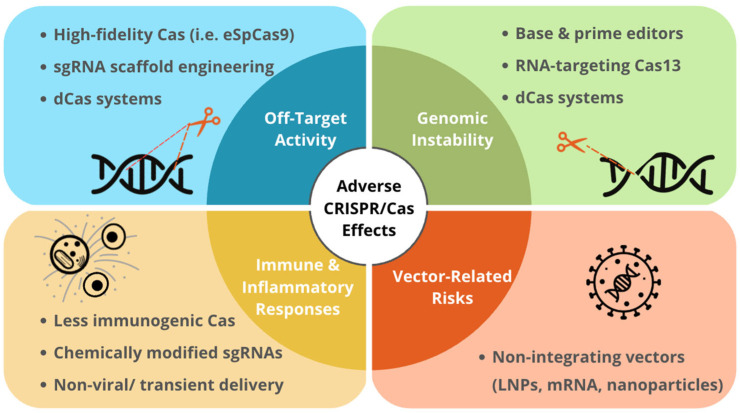
Long-Term Effects of Expression of CRISPR/Cas Systems and the Corresponding Modifications.

The major long-term adverse effects associated with CRISPR/Cas therapies and corresponding molecular modifications to address these challenges are summarized in Figure 1 [14,23,97].

Summary of studies evaluating long-term effects of expression of CRISPR/Cas systems and the corresponding technical development in different biological models. 

## 6. Long-Term Effects of Expression of CRISPR/Cas Systems in In Vitro Models, In Vivo Models, and Humans

**Figure 2:**
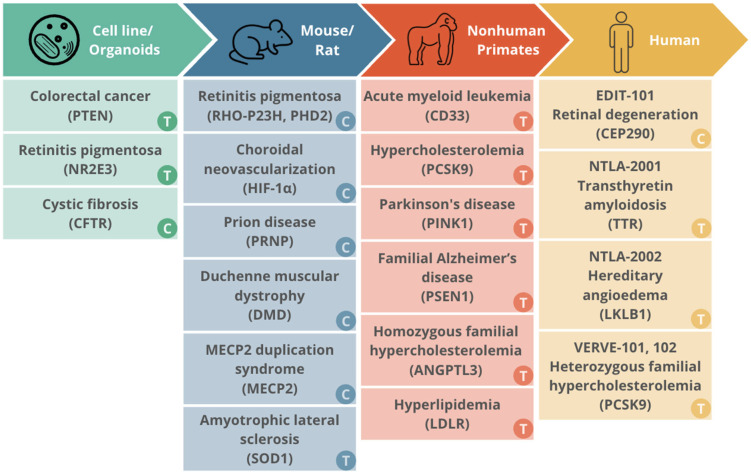
The translational landscape of CRISPR/Cas trials from in vitro models to human clinical trials.

In order to advance CRISPR/Cas systems as an avenue for gene therapy, it is necessary to understand the long term effects of their expression. Sustained Cas expression is often necessary to achieve high editing rates, particularly in post-mitotic tissues. Studies in in vitro models, rodents, non-human primates, and humans have begun to demonstrate the feasibility and safety of longer term Cas expression, with encouraging therapeutic results as well as limited off-target consequences. Below, we describe selected studies in model systems and humans, with a focus on more durable modes of delivery (e.g., viral versus LNP) and longer study follow-up periods in the span of weeks to months. Figure 2 summarizes studies evaluating chronic or long-term effects of expression of CRISPR/Cas expression systems in different biological models [50,98,99,100,101,102,103,104,105,106,107,108,109,110,111,112,113,114,115,116,117,118,119,120,121,122,123,124,125,126,127,128,129,130,131,132,133,134,135,136,137,138,139,140].

Summary of the progressive translation of CRISPR/Cas therapeutic platforms across different experimental models, from initial validation in cell lines and organoids to human clinical trials.

### 6.1. Cell Line and Organoid Models

Extensive research has also explored the long-term effects of CRISPR/Cas activity in cell and organoid models. Organoids have bridged the gap between in vivo tissue-based observations and those made in cell lines cultured in two dimensions. These systems allow continuous expression and monitoring of Cas nucleases under controlled conditions, making them valuable for evaluating cumulative off-target effects, genomic stability, and potential cellular adaptations over extended periods. Key studies using cell lines and organoids are summarized in Table S1.

Research utilizing transient delivery has demonstrated durable therapeutic effects through precise genomic modifications. Skoufou-Papoutsaki et al. (2023) [106] employed RNP-based CRISPR targeting the PTEN tumor suppressor gene in patient-derived intestinal organoids. to investigate the consequences of PTEN deficiency. Editing efficiency reached up to 98%, and off-target effect only found in a 100% homologous sequence. Similarly, Diakatou et al. [52] knock out the G56R mutation in nuclear receptor subfamily 2 group E member 3 (NR2E3) for retinitis pigmentosa (RP), using eSpCas9. The gene editing could maintain genetic stability and pluripotency throughout the differentiation into retinal organoids without detectable off-target events.

In contrast, other studies have evaluated sustained Cas presence to achieve correction of complex mutations. Bulcaen et al. [107] applied prime editing to correct *CFTR* mutations in patient-derived rectal organoids for cystic fibrosis (CF). By using lentiviral vectors for delivery, they achieved up to 34% genomic correction and 80% functional rescue. Notably, even after prolonged high-level exposure to the prime editing components, no activity was detected at predicted off-target sites. 

These findings suggest that while transient delivery is a plausible and often preferred method for single-course genomic correction due to its reduced exposure window, sustained expression via viral vectors can be effectively managed and monitored in organoid systems to confirm long-term safety and functional recovery.

### 6.2. Murine Models

Extensive research has also been conducted to examine the toxicity, immunogenicity, and oncogenic risk of CRISPR-based genome editing in mouse and rat models. Mice and rats share substantial physiological and genetic homology with humans and are easy to breed and maintain with rapid reproductive cycles, making them a suitable model for initial investigation of Cas nuclease expression. Key murine studies involving in-tissue editing with study periods in the range of several months to years are described below, with key study characteristics and outcomes summarized in Table S1.

A proline-to-histidine substitution at codon 23 of *rhodopsin* (*Rho*) is the most common autosomal dominant RP mutation. Shahin et al. 2022 found that subretinal AAV8 vectors expressing SaCas9 and a P23H-specific sgRNA disrupted the mutant *Rho* allele in 5.97% homozygous mutants and 14.8% and 12.08% of photoreceptors in two heterozygous rat lines [108]. Treated eyes retained normal electroretinogram waveforms and demonstrated improved visual acuity compared to untreated contralateral eyes for ≥ 450 days. Furthermore, deep sequencing of eight Cas-OFFinder sites revealed no off-target indels, demonstrating durable efficacy and retinal safety for this duration of SaCas9 expression in the rat model [108]. Other studies have demonstrated successful knockdown of C110R and T17M autosomal dominant with follow-up ranging from 6 to 11 months as well as correction of the autosomal recessive E150K mutant allele with no significant off target effects [109,110,111,112]. Another study found that knockdown of the *Nrl* gene in vivo was also able to rescue retinal degeneration in *Rho* P347S mice with no observed off-target mutations at 10 genomic loci when followed for 9.5 months [111]. 

RP is characterized not only by genetic heterogeneity across >80 genes and 3100+ mutations, but also by shared metabolic dysfunction independent of the causal mutation. To address this, Nolan et al. 2024 developed a gene-agnostic CRISPR-based therapy targeting the glycolytic metabolome [113]. They used AAV-delivered CRISPR/Cas9 to ablate *PHD2* (*prolyl hydroxylase domain-containing protein 2*) in rod photoreceptors of two independent RP mouse models: a dominant *Rho^P347S^* model and a recessive *Pde6b^rd10^* model. The therapy stabilized hypoxia-inducible factors (HIFs) and rejuvenated aerobic glycolysis specifically in diseased rods. At 4 weeks post-treatment, both models showed preserved retinal structure on optical coherence tomography and maintained electroretinogram responses. Histological analysis revealed preserved photoreceptor nuclear layers compared to controls. Importantly, editing efficiencies reached 60–70% in rods with minimal off-target effects detected by deep sequencing. No adverse events were observed during the follow-up period. This metabolic reprogramming approach represents a paradigm shift toward mutation-independent therapies for genetically heterogeneous diseases [113].

Hypoxia-inducible factor 1α (HIF-1α) is an intracellular protein involved in the detection of hypoxic conditions that is a target for suppressing choroidal neovascularization. Jo et al. 2018 used an adeno-associated virus serotype 9 (AAV9) vector to deliver Campylobacter jejuni Cas9, the smallest well-characterized Cas9 ortholog, and a U6-driven sgRNA targeting *HIF-1α* into the mouse vitreous cavity of the retina via injection [114]. Editing efficiencies ranged from 49% to 75%, with no functional toxicity detected on electroretinogram, an electrophysiological assay for retinal function. Fourteen months after treatment, on-target editing rates further increased, while deep sequencing of seven off-target candidates identified by Cas-OFFinder revealed indel frequencies at background levels [114].

Prion diseases are a group of fatal neurodegenerative disorders caused by the misfolding of the prion protein (PRNP). An et al. 2025 used an AAV vector to deliver a cytosine base editor and an sgRNA designed to introduce a premature stop codon in *PRNP* into the central nervous system of a humanized mouse model at 6–9 weeks of age [115]. Mice were followed for up to 600 days. Treated animals exhibited prolonged survival compared to controls and maintained steady weight gain, whereas untreated mice experienced progressive weight loss. In mice at the 600-day time point, off-target editing was observed at eight loci, with higher editing levels than in mice evaluated at 35 and 100 days. However, testing for off-target editing with the same CRISPR/Cas system in human cells revealed that off-target sites were primarily located in intergenic regions with no candidate off-target sites associated with tumor suppressor genes. Furthermore, no unintended editing was detected in the liver or any transgene expression in the dorsal root ganglion, demonstrating tissue specificity [115].

Duchenne muscular dystrophy (DMD) is a severe muscle-wasting disease caused by mutations in the *DMD* gene, which encodes dystrophin. Mutations are heterogeneous with point mutations accounting for 10% of cases [116]. Xu et al. 2021 engineered an adenine base editor targeting a premature *DMD* stop codon packaged in an AAV9 vector and delivered via intravenous tail injection to mdx^4cv^ mice at 5 weeks of age [117]. At 10 months post-treatment, dystrophin protein was nearly fully restored in the heart (~96%) and partially in skeletal muscle (~15%), resulting in improved muscle histology and function. No liver toxicity, tested by checking for elevated aspartate aminotransferase and alanine aminotransferase levels in the treated mice, or significant off-target editing in other regions of the genome was found. However, some bystander editing at the mdx^4cv^ locus and transcriptome editing was observed, which may demonstrate a need for higher specificity [117].

Methyl-CpG-binding protein 2 (MECP2) duplication syndrome (MDS) causes severe neurological symptoms due to extra copies of *MECP2*. Yang et al. 2025 designed AAV-delivered Cas13-based RNA editors with a sgRNA targeting *MECP2* and injected the system into the lateral ventricle of a postnatal day 1 mouse model for MDS [118]. The mice were followed for 4 weeks and demonstrated improved motor, exploratory, and social behavior and decreased anxiety compared to deficits present in the untreated mouse model. To examine toxic and immunogenic effects, the researchers assessed liver toxicity, inflammatory cytokines, and injury biomarkers and found no changes in levels compared to the untreated mice [118].

Together, these studies underscore the potential of CRISPR-based genome editing for durable, tissue-specific therapeutic outcomes in rodent models of human disease. Across diverse targets, including the retina, muscle, central nervous system, and liver, expression of Cas nucleases and RNA-targeting enzymes through viral delivery systems demonstrated sustained efficacy with minimal off-target activity and acceptable safety profiles. While certain challenges, such as low levels of off-target editing, transcriptomic effects, and structural rearrangements were observed, these findings largely support the feasibility of durable CRISPR expression for in vivo therapeutic applications.

### 6.3. Nonhuman Primate Models

Compared with mice, nonhuman primates share greater genetic and phenotypic similarity with humans, which makes them a suitable model for studying the potential outcomes of genome editing in humans [119]. Moreover, nonhuman primates have longer lifespans than rodents, enabling multi-year studies to assess the long-term safety and efficacy of gene editing therapies. While many therapies utilize transient delivery methods to achieve permanent results, such as LNP and RNP systems, it remains unknown whether even brief exposure to these components may induce delayed or chronic biological effects over a patient’s lifetime.

Key studies involving gene editing with long follow-up study periods were described below with key study characteristics and outcomes summarized in Table S1.

Research utilizing transient delivery (short-term Cas expression) has demonstrated durable therapeutic effects. For instance, CRISPR/Cas9 delivered as RNP complexes achieved stable, multilineage engraftment in *CD33* knockout models for Acute myeloid leukemia [120,121]. Similarly, LNP-based delivery of Adenine Base Editor targeting *PCSK9* resulted in a 65% reduction in low-density lipoprotein cholesterol (LDL-C) that remained durable for years despite the rapid clearance of the nuclease, with minimal off-target effect (<1%) [122,123]. Beyond systemic delivery, physical transient methods such as microinjection have also proven effective for long-term modeling. Chen et al. (2021) [124] used CRISPR-Cas9-D10A nickase microinjection to target Exon 2 of *PTEN-induced kinase 1* (PINK1), associated with early-onset Parkinson’s disease, in cynomolgus monkey embryos, achieving an overall editing efficiency of 45.5%, with indel frequencies ranging from 9.1% to 100% per embryo with no off-target effects or impaired embryonic development. Likewise, Li et al. (2024) applied a dual-guide CRISPR/Cas9 system to permanently excise *PSEN1* exon 9, a common cause of familial Alzheimer’s disease, demonstrating high efficiency (76.5% of zygotes) and minimal off-target effects [125].

Among non-human primate studies, some are notable because their therapeutic platforms have already advanced to human trials. Although details are limited, these studies demonstrate durable editing, favorable safety, and provide essential translational evidence for first-in-human applications.

VERVE-201, that uses GalNAc-conjugated lipid nanoparticles (GalNAc-LNPs) to deliver a base editor targeting the *angiopoietin-like 3* (*ANGPTL3*) gene in the liver, aims to reduce LDL-C and remnant cholesterol. A Phase 1b trial (PULSE-1, NCT06451770) is currently evaluating its safety and tolerability in adults with RH. In a long-term study of 34 nonhuman primates [126], VERVE-201cyn, achieved durable gene editing (up to 63%) and sustained protein reductions (up to 96%) for at least 22 months without evidence of long-term toxicity. Editas Therapeutics reports that one of their in vivo therapies, EDIT-401, edits the LDLR gene to reduce LDL-C levels with the aim of treating hyperlipidemia in patients. Pre-clinical studies in non-human primates have shown a ~90% mean reduction of LDL-C and in safety measures, there were no adverse events reported; transient increases in liver enzyme biomarkers were observed, which resolved within one week [105]. These studies provide essential translational evidence that a single course of transient nuclease exposure can suffice for lifelong therapeutic benefit, offering a potentially safer alternative to the continuous nuclease activity found in AAV-mediated more durable expression systems. 

### 6.4. Humans

While ex vivo modification by CRISPR/Cas9 systems has been used therapeutically, here we highlight in vivo interventions using CRISPR/Cas9 therapies in several organ systems, with particular emphasis on outcomes observed during extended clinical follow-up. Key study characteristics and outcomes are summarized in Table S2.

When evaluating in vivo CRISPR/Cas9 therapies, the immune landscape of the targeted organ system plays a critical role in determining the likelihood and severity of an immune response. Certain anatomical sites in the human body–such as the eye, central nervous system, placenta and fetus, and testes–are classified as immune privileged [127]. These regions have evolved mechanisms to limit inflammatory and immune-mediated responses to foreign antigens, which may mitigate immunogenicity and support favorable outcomes during prolonged post-treatment observation. Leveraging such immunological features may improve the durability and safety of in vivo gene-editing strategies.

The eye provides a well-characterized example of how anatomy and immune regulation can support sustained therapeutic benefit. The subretinal space of the eye is physically separated from systemic circulation by the blood-retina barrier, allowing it to serve as a relatively isolated environment [128]. This makes it an ideal space for localized CRISPR delivery when treating inherited retinal diseases. The separation reduces exposure to systemic immune surveillance and may lower the risk of immune-mediated complications over time. Indeed, research comparing vitreous fluid biopsies to serum samples has found that pre-existing immunity to Cas9 may represent a lower risk in the ocular compartment than systematically [129]. These strategies are also utilized by other optogenetic therapies including Nanoscope’s MCO-010 which reprograms bipolar cells into light-sensitive cells, offering an alternative pathway when rods and cones are damaged in inherited retinal diseases like retinitis pigmentosa [130]. Similar considerations apply to other immune-privileged sites, where reduced immune activation may provide a strategic advantage for CRISPR-based treatments.

The first clinical trial of in vivo CRISPR/Cas9 expression in humans was initiated as part of the BRILLIANCE Trial (NCT03872479) of EDIT-101, a gene therapy developed to treat a specific type of CEP290-associated inherited retinal degeneration, a condition causing severe early-onset vision loss. EDIT-101’s CRISPR/Cas9 system is designed to permanently remove the pathogenic *CEP290* IVS 26 variant. The trial sponsored by Editas Medicine, Inc. began in 2019 and reported the two-year interim results in 2024 [131]. EDIT-101 was administered in a phase 1–2 open-label, single ascending dose study in 12 adults (17–63 y/o) and 2 pediatric (9 and 14 y/o) participants with LCA10-IVS26 [132]. No serious adverse events related to the treatment or procedure and no dose-limiting toxic effects were recorded. Six participants demonstrated meaningful improvement in cone-mediated vision as assessed with FST and six participants had a meaningful improvement from baseline in the vision-related quality of life score. After three months, however, only three of the 14 participants had BCVA improvement that reached the prespecified threshold for clinically meaningful improvement of at least 0.3 logMAR. The researchers concluded that subretinal delivery of EDIT-101 had a favorable safety profile and provided proof of concept for in vivo CRISPR/Cas9 gene-editing in inherited retinal disorders. Editas’s approach to treat *CEP290*-LCA10, which relies on an AAV5 vector to drive persistent expression of gRNAs and Cas9, was deemed safe for clinical application by the FDA [133]. Subsequent reporting in the *New England Journal of Medicine* details results from their Phase I/II clinical trial wherein 12 adults and 2 children were subretinally injected with EDIT-101 [132]. The median follow-up period was over a year and up to 454 days. Across this follow-up period, no adverse effects attributable to Cas9 activity were observed, with reported finding limited to surgery-related effects. Although Editas Medicine has since paused further development of EDIT-101, these data remain an important benchmark for understanding the longitudinal safety of in vivo CRISPR/Cas therapies. As of September 2025, the company is now developing another in vivo medicine, EDIT-401, to target hyperlipidemia and atherosclerotic cardiovascular disease, with human proof-of-concept data anticipated by the end of 2026 [97].

The genetic complexity of inherited retinal diseases further underscores the need for both gene-specific and mutation-agnostic CRISPR therapies. Kolesnikova et al. 2023 characterized the phenotypic variability in 11 patients with biallelic variants in *CLN* genes (*CLN3*, *CLN7/MFSD8*, *CLN8*, and *GRN/CLN11*), which cause neuronal ceroid lipofuscinoses (NCLs) and isolated retinal dystrophies [134]. Five novel variants were identified. Among patients with *CLN3* variants, five presented with classic juvenile NCL (Batten disease) with rapid vision loss (onset age 6–8 years) followed by neurological symptoms (onset age 7–12 years), while one patient had isolated retinitis pigmentosa without neurological involvement at age 31. Patients with *MFSD8* variants presented with cone-rod dystrophy (CORD) with onset at ages 5–13.5 years. Full-field electroretinography revealed extinguished or severely reduced responses, and OCT imaging showed diffuse retinal thinning with loss of ellipsoid zones. The substantial phenotypic variability—even among patients with identical alleles—highlights the challenges in establishing genotype-phenotype correlations and emphasizes the importance of comprehensive patient databases for counseling. This variability reinforces the rationale for developing both allele-specific CRISPR knockout strategies (as demonstrated in the Diakatou NR2E3 study) and gene-agnostic metabolic reprogramming approaches (as shown in the Nolan and Caruso studies) to address the full spectrum of inherited retinal diseases [134].

Longer-term follow-up has also been reported in non-ocular indications. A clinical trial investigated the safety and efficacy of BD111, CRISPR/Cas9 mRNA therapy, delivered via corneal injection for refractory herpetic viral keratitis (NCT04560790), followed three participants for up to 21 months [135]. Although results remain unpublished beyond a 2023 preprint, investigators reported no viral relapse, disease recurrence, or CRISPR-associated adverse effects during extended follow-up. This study was the first to investigate in vivo CRISPR therapy for treating infectious diseases and the first virus-like particle-delivered gene therapy. For 18 months on average in all three patients, they observed neither detectable CRISPR-induced off-target cleavages nor systemic adverse events. 

In systemic indications, Intellia Therapuetics has reported longitudinal data from multiple in vivo CRISPR/Cas 9 programs. NTLA-2001 (nexiguran ziclumeran, nex-z), targets misfolded transthyretin protein (TTR) into six patients (46–64 y/o) with hereditary transthyretin amyloidosis with polyneuropathy (hATTRv-PN) and patients with transthyretin amyloidosis-related cardiomyopathy (hATTRv-CM) (NCT04601051). The trial began in 2020 and researchers reported interim 28-day results in 2021 and 12-month results in 2024 [55,136]. After 28 days, three of the six patients reported mild adverse events, but no serious adverse events were reported. At day 28, each patient had sustained reductions in the serum TTR protein concentration. After twelve months of follow-up, 36 patients received NTLA-2001. In these patients, the mean percent change from baseline in the serum TTR level was −89% (95% confidence interval [CI], −92 to −87) at 28 days and −90% (95% CI, −93 to −87). Serious adverse events were reported in 14 of the 36 patients, with 7 reported to have had a serious adverse event that led to hospitalization associated with cardiac failure (in 4 patients), arrhythmia (in 2 patients), or both (in 1 patient) [136]. The investigators noted that these serious adverse events were consistent with patients and only considered one of these events to be related to NTLA-2001 (a serious infusion reaction). The study is estimated to be completed in August of 2025 and the company expects to present longer-term data from the Phase 1 portion in the second half of 2025 [137]. Researchers have since also begun two separate phase 3 trials, MAGNITUDE (NCT06128629) which specifically investigates the efficacy and safety of NTLA-2001 in patients with hATTRv-CM and MAGNITUDE-2 (NCT06672237) for patients with hATTRv-PN. Primary completion is estimated for December 2027. 

Intellia Therapeutics has also reported the longest follow-up data after in vivo CRISPR/Cas expression that is currently public. The company is sponsoring a phase 1–2 trial of NTLA-2002 (lonvoguran ziclumeran, lonvo-z) to treat hereditary angioedema, a condition characterized by severe, recurring and unpredictable inflammatory attacks in various organs and tissues of the body (NCT05120830). NTLA-2002 is a one-time in vivo CRISPR/Cas9-based therapy designed to inactivate KLKB1, which encodes the precursor protein prekallikrein. Results of three-year follow-up data from the phase one portion were shared in an oral presentation at the European Academy of Allergy and Clinical Immunology (EAACI) Congress held 13–16 June 2025, with a data cutoff of 12 February 2025. There, they reported that all 10 patients from the phase 1 portion were free from both hereditary angioedema attacks and chronic therapy at nearly two years of median follow-up. Across all three dose levels, the intervention was well tolerated and continued to demonstrate a favorable safety profile with the most frequent adverse event reported being infusion-related reactions. As such, Intellia is proceeding with a phase 3 trial, HAELO, to assess the safety and efficacy of the intermediate, 50 mg dosage [136]. 

Verve Therapeutics similarly emphasized longitudinal assessment in its development of in vivo base-editing therapies targeting PCSK9. VERVE-101 and VERVE-102 are one-time therapies that use an adenine base editor and guide RNA designed to inactivate PCSK9 to reduce LDL-C in patients with cardiovascular disease. During the phase 1 trial of VERVE-101 one of the six patients treated with a 0.45 mg/kg dose of VERVE-101 experienced grade 3 increases in a particular liver enzyme and a grade 3 case of low blood platelets. The patient was hospitalized and observed for two days despite being asymptomatic. The individual experienced no bleeding, and the lab abnormalities resolved independently within a few days [138,139]. VERVE-102 incorporates an alternative ionizable lipid and N-acetylgalactosamine (GalNAc) targeting, a difference that highlights the importance of delivery methods in the efficacy and safety profiles of these therapies. VERVE-102’s Heart-2 Phase 1b clinical trial was conducted in 14 patients with heterozygous familial hypercholesterolemia and premature coronary artery disease (CAD), and initial data were announced in April of 2025 [140]. Researchers reported initial mean blood LDL-C and PCSK9 protein reductions from baseline, using a time-averaged percent change from day 28 through the last available follow-up. They showed that a single infusion led to dose-dependent decreases in the blood of PCSK9 and LDL-C (LDL-C mean reduction of 53% in the four patients of the 0.6 mg/kg dose cohort, 21% of the four 0.3 mg/kg-dose participants, and 41% of the six 0.45 mg/kg-dose participants). They also report no treatment-related serious adverse events and expect to report final dose escalation data in the second half of 2025. 

Collectively, these trials demonstrate a promising trajectory for in vivo CRISPR/Cas therapeutics across diverse indications. While there are alternative existing therapies for many of these conditions, many do not target the specific gene causing the condition and do not cure the patient of disease (Table 3). In parallel, ex vivo CRISPR/Cas therapies-such as Casgevy, approved December 2023 by the FDA for sickle cell disease and transfusion-dependent β-thalassaemia-continue to expand the clinical footprint of genome editing [141].

Importantly, across multiple studies–EDIT-101, NTLA-2001, NTLA-2002, and VERVE-102–researchers have reported encouraging safety profiles, absence of serious adverse events, and disease-specific improvements in clinical outcomes. Together, these findings provide crucial proof-in-concept for the therapeutic viability of one-time, in vivo CRISPR/Cas therapies, especially when paired with strategic delivery methods and targeting immune-privileged or localized tissues. These advances, however, also highlight a need for long-term monitoring, larger cohort validation, and careful assessment of immunogenicity, off-target effects, and durability of gene edits. While many of these therapies remain in early-phase clinical trials, the initiation of multiple Phase 3 trials signals a growing confidence in CRISPR/Cas systems’ growing potential. 

## 7. Outlook and Conclusions

The field of CRISPR/Cas gene-editing is making steady progress toward safe and effective therapeutic applications, supported by strong evidence from preclinical studies through clinical trials. Early research in rodent models established foundational knowledge about delivery methods, precision, and initial safety profiles, which paved the way for studies in more clinically relevant systems. Nonhuman primates have provided important insights into the long-term stability of gene edits and immune responses over several years. These studies show that CRISPR therapies can produce lasting gene changes without serious side effects, building confidence as researchers work on treating complex, long-lived tissues and systemic diseases.

In human trials, targeting immune-privileged sites and improving delivery methods have been key to reducing immune reactions and improving treatment outcomes. Successful treatments for inherited retinal diseases and systemic conditions like transthyretin amyloidosis and hereditary angioedema demonstrate both the flexibility of CRISPR technologies and their ability to adapt to different clinical needs. Favorable safety profiles, along with sustained improvements in function and reduced disease markers, highlight CRISPR’s transformative potential. Thus far, longer term trials in murine, non-human primates, and humans have demonstrated minimal to no evidence of off-target editing. Among the available data, only one murine study reported large deletions in the range of hundreds to thousands of base pairs. No indels were detected in non-human primate trials and the one human study that performed sequencing likewise reported no off-target editing. Furthermore, to date, no gain-of-function mutations that could promote clonal expansion or tumorigenicity have been identified in CRISPR therapeutics. However, there remains a critical need for ongoing, long-term monitoring in treated patients to assess potential late–emerging effects, including immune responses, off-target edits, and oncogenic risk–especially as treatments expand beyond immune-protected areas and into broader clinical use. 

Looking ahead, the field needs to address important gaps, including longer-term studies of newer gene-editing tools such as base and prime editors, and acquire a deeper understanding of the effects of sustained Cas protein expression in humans. Continued innovation in delivery methods will be essential to reach a wider range of tissues and diseases. Overall, combining preclinical research, careful clinical trials, and continuous technological improvements sets a strong path for CRISPR/Cas therapies to become a key part of precision medicine—offering new ways to treat genetic diseases while balancing effectiveness and safety over a patient’s lifetime through rigorous and sustained post-treatment surveillance.

## Figures and Tables

**Table 1:** Comparison between transient and sustained delivery systems.

Feature	Transient Delivery	Sustained Delivery
Active Duration	Hours to Days	Months to Permanent,
Primary Vehicles	mRNA, RNPs, AdVs,	Integrating LVs, episomal AAV,
Off-Target Risk	Lower (limited active time)	Higher (prolonged activity),
Immunogenicity	Generally lower/transient	Potentially persistent/toxic

**Table 2:** Summary of Long-Term Adverse Effects of Chronic CRISPR/Cas Expression.

Adverse Effect	Mechanism	Consequences	Modifications to Address Adverse Effects
Double Stranded Breaks (DSB)	CRISPR/Cas9 endonuclease activity forms DSBs	Indels Chromosomal translocations	Base editors and prime editors that do not create DSBs Cas13 RNA editor instead editing DNA
Off-Target Genomic Editing	Nonspecific sgRNA binding	Editing at other loci	High fidelity Cas enzymes sgRNA design
Off-Target Transcriptomic Effects	Off-target base editing Off-target transcription repressor-fused dCas activity	Unintended changes in gene expression	High fidelity Cas enzymes sgRNA design
Genomic Vector Integration	Integration of AAVs into host genome	Introduction of new mutations Low oncogenic risks	Alternative delivery vectors Non-DSB editing approaches
Immunogenicity	Cas protein sgRNAs Delivery vector	Elevated inflammatory markers Hyperactivation of the innate immune system, hemolytic disorders at high doses of AAVs	Alternative delivery vectors Engineering less immunogenic sgRNAs
Tumorigenicity	DSBs Off-target editing	Activation of oncogenic genes or suppression of tumor repressor genes	Modifications reduce DSBs and off-target editing

**Table 3:** Existing therapies for conditions treated in human CRISPR/Cas clinical trials.

Drug Name	Condition Treated	Alternative Therapies
EDIT-101 [136]	CEP290-associated inherited retinal degeneration (Leber’s Congenital Amaurosis)	MCO-010 * [142], GS030 * [143], QR-110 [144]
NTLA-2001 [55]	ATTR amyloidosis	Patisiran [145], Vutrisiran [146], Inotersen [147], Eplontersen [148]
NTLA-2002 [141]	Hereditary Angioedema (KLKB1 target)	Donidalorsen [149], Garadacimab * [150], BMN-331 [151]
VERVE 102 [145]	LDL-cholesterol excess (PCSK9 target)	VERVE-201 [152], CTX310 [153], Inclisiran [154], RN0191 [155], ARO-ANG3 [156], Vupanorsen [157]

Specific information on each therapeutic can be found in Supplemental Table S2. * denotes gene-agnostic therapies.

## Data Availability

The original contributions presented in this study are included in the article/Supplementary Material. Further inquiries can be directed to the corresponding author(s).

## Supplementary Materials

The following supporting information can be downloaded at: https://www.mdpi.com/article/10.3390/cells15020156/s1, Table S1: Key outcomes and characteristics of long term expression of CRISPR/Cas in human trials, Table S2: Additional information about alternative therapies for conditions treated in CRISPR/Cas human trials.

## Abbreviations

The following abbreviations are used in this manuscript:

CRISPR	clustered regularly interspaced short palindromic repeats
Cas	CRISPR-associated
crRNA	CRISPR RNAs
PAM	protospacer adjacent motif
gRNA	guide RNA
sgRNA	single-guide RNA
tracrRNA	trans-activating CRISPR RNA
DSB	double strand break
NHEJ	non-homologous end joining
HDR	homology-directed repair
HEPN	higher eukaryotes and prokaryotes nucleotide-binding
ssRNA	single-stranded RNA
AAV	adeno-associated virus
LNP	lipid nanoparticle
RNP	ribonucleoprotein complexes
GalNAc	N-acetylgalatosamine
Ku	Ku70-Ku80
DNA-PKcs	DNA-dependent protein kinase catalytic subunit
MMEJ	microhomology mediated end joining
SSA	single stranded annealing
indels	insertions and deletions
CRISPRi	CRISPR interference
eSpCas9	enhanced specific SpCas9
dCas9	dead Cas9
CRISPRa	CRISPR activation
SpCas9	*Streptococcus pyogenes* Cas9
SaCas9	*Staphylococcus aureus* Cas9
dCas13	dead Cas13
m^6^A	N^6^-methyladenosine
PTEN	phosphatase and tensin homolog deleted on chromosome ten
RP	retinitis pigmentosa
NR2E3	nuclear receptor subfamily 2 group E member 3
iPSC	induced pluripotent stem cells
CF	cystic fibrosis
CFTR	cystic fibrosis transmembrane conductance regulator
AAV9	adeno-associated virus serotype 9
HIF-1α	hypoxia-inducible factor 1α
Rho	rhodopsin
PRNP	prion protein
DMD	Duchenne muscular dystrophy
MECP2	Methyl-CpG-binding protein 2
MDS	MECP2 duplication syndrome
ALS	amyotrophic lateral sclerosis
SOD1	superoxide dismutase 1
AML	acute myeloid leukemia
CD33	sialic acid-binding Ig-like lectin 3
CVD	cardiovascular disease
LDL-C	low-density lipoprotein cholesterol
ABE	adenine base editor
PCSK9	proprotein convertase subtilisin/kexin type 9
PD	Parkinson’s disease
PINK1	PTEN-induced kinase I
FAD	familial Alzheimer’s disease
PSEN1	presenilin 1
GalNAc-LNP	GalNAc-conjugated lipid nanoparticle
ANGPTL3	angiopoietin-like 3
LDLR	low density lipoprotein receptor
TTR	transthyretin protein
